# How are health workers paid and does it matter? Conceptualising the potential implications of digitising health worker payments

**DOI:** 10.1136/bmjgh-2021-007344

**Published:** 2022-01-25

**Authors:** Margaret McConnell, Mansha Mahajan, Sebastian Bauhoff, Kevin Croke, Stéphane Verguet, Marcia C Castro, Kheya Melo Furtado, Abha Mehndiratta, Misha Farzana, Sabina Faiz Rashid, Richard Cash

**Affiliations:** 1 Global Health and Population, Harvard University T H Chan School of Public Health, Boston, Massachusetts, USA; 2 Goa Institute of Management, Ribandar, Goa, India; 3 BRAC University James P Grant School of Public Health, Dhaka, Dhaka District, Bangladesh

**Keywords:** health systems, health policy, health economics

**Summary box**

- Payment digitisation efforts in the health sector in low/middle-income countries have accelerated due to the COVID-19 pandemic.
- Research on impacts of worker payment digitisation on health systems is lacking.
- Our conceptual model details how payment digitisation could improve health systems.
- Wage digitisation has the potential to improve health system performance and provider well-being and consequently, patient outcomes.
- Critical gaps in evidence need to be addressed to support implementation and effective innovation.

Health systems are striving to improve their effectiveness in response to evidence of significant avertable mortality stemming from challenges in the delivery of high quality healthcare.^1^ Ensuring that facilities are staffed with well-trained, motivated, fairly compensated health workers is a major challenge, yet paramount to improving health system performance and achieving universal health coverage.^2–4^ Beyond the health facility, frontline community health workers (CHWs) in many low/middle-income countries (LMICs) are charged with the essential task of communicating key health and programmatic information to communities in a culturally sensitive manner, while in many cases receiving limited formal compensation.

Increasing evidence suggests that many health workers lack critical knowledge of key guidelines and procedures,^5–7^ and do not always adhere to guidelines even when they demonstrate knowledge.^5^ Absenteeism and turnover of trained providers undermines quality of care and the delivery of life-saving interventions.^8^ Health workers perform more of the tasks they know how to do when they are working in private facilities,^5^ suggesting a potential link between payment and performance. Yet the link between motivation, retention, compensation and performance is complex.^4 9^ The amount and design of supplemental incentives have been a major focus of health sector reforms in LMICs,^10^ with mixed evidence of impact and effectiveness.^11^ However, the prevalence of payment system failures, such as inefficiencies in the documentation and verification of work, delays in payment, frictions in accessing payments and leakage of resources, has not been a central focus of inquiry. These payment challenges have the potential to affect health workers at all levels of the health system but may be most pronounced among vaccination campaign workers or CHWs. These workers are generally not salaried civil servants and instead are typically hired on a temporary basis for campaigns or considered volunteers who receive some compensation. As a result, there are fewer standardised payment protocols and a greater likelihood of cash-based payments for these workers. In many LMICs, these frontline lower level health workers are overwhelmingly likely to be women.

Other sectors in LMICs, including education and social protection, have faced challenges with payments that undermine the performance of frontline workers and the efficiency of service delivery. With the global expansion of digital financial services, public sectors in some countries are moving towards digitising the payments of frontline workers. While the expansion of digital payments in the health sector had lagged efforts in other sectors, the transition from cash to digital payments in the health sector has accelerated recently due to the COVID-19 pandemic. Efforts are underway to digitise the payments of vaccination campaign workers by the WHO for polio campaigns, by GAVI for immunisation campaigns, and by the Global Fund for Aids, Tuberculosis and Malaria. In a survey of 382 vaccinators in 113 districts after a polio vaccination campaign in Côte d’Ivoire in 2020, where campaign workers were paid using digital mobile money transfers, 83% of the workers reported that they would prefer digital payments to cash payments in future campaigns.^12^

Although there is increasing focus on payment digitisation, including for lower level health workers (such as vaccination campaign workers and CHWs), there is limited rigorous evidence on the impacts of digitising health worker payments on health system outcomes.^13^ Moreover, while digitisation of payments in other sectors has been studied with large-scale experimental approaches demonstrating some benefits,^14^ rigorous research is needed within the health sector. This evidence would help policymakers understand whether payment digitisation reaches its desired objectives of improving health system performance, as well as the extent to which payment digitisation may generate unintended consequences. Research designs to evaluate the impact of digital payments could be informed by implementation research embedded within ongoing efforts to rollout digitisation of health worker payments.

Efforts to improve health worker payment processes have largely focused on digitising payments through bank transfers or mobile money payments. A better understanding of other bottlenecks to payments could facilitate further improvements in payment processes, including integration into m-health efforts to digitise and standardise processes of documentation and verificiation of work. In order to develop this research agenda, a conceptual understanding of the potential impacts of digitising payments on health system performance, provider well-being and patient outcomes is needed. In figure 1, we outline a conceptual model of the potential impacts of payment digitisation in the health sector.

**Figure 1:**
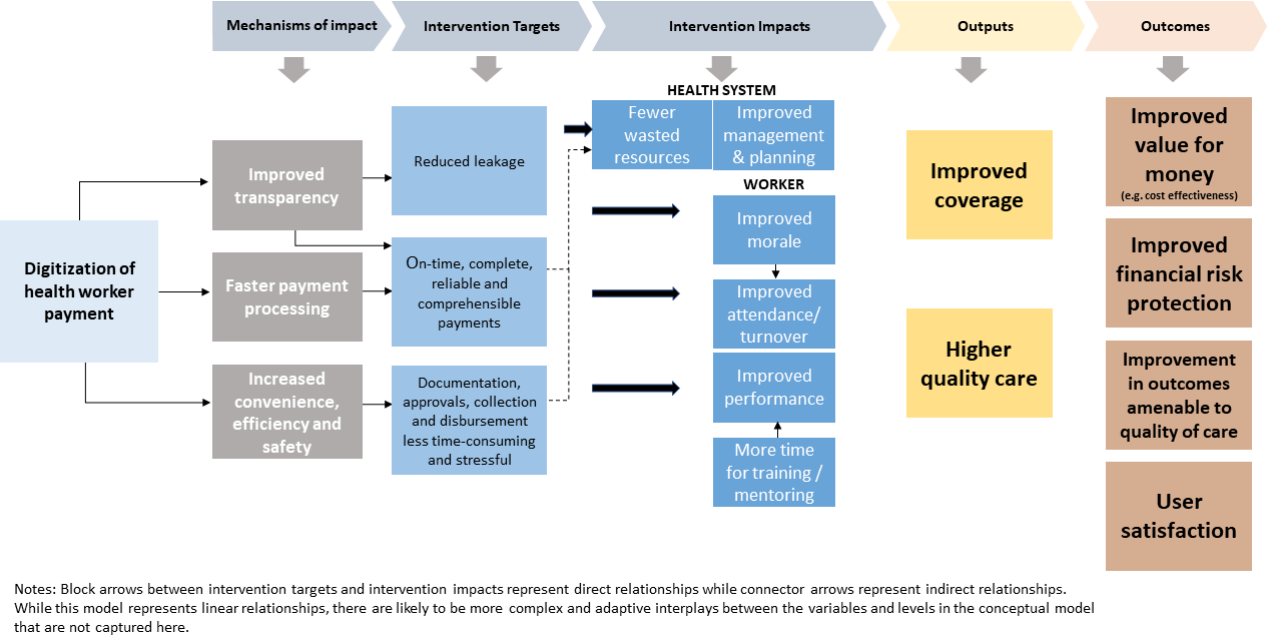
Conceptual model of the potential impacts of health worker wage digitisation.

First, digital payments may strengthen accountability, particularly when compared with cash payments. Digital payments could improve the ability to track where funds are spent and reduce leakage, so that intended beneficiaries receive the full value of the funds they are due.^15^ In order to process payments, workers and health facilities must report hours worked and receive verification from multiple levels of management. Fragmented systems for documentation of work and payment can also result in out-of-date rosters of health workers where payments are allocated to ‘ghost’ workers, resulting in leakage of resources out of health system budgets.^16^ By some estimates 41%–80% of health resources leak out of the health system without appearing as health expenditures.^17^ Although technology itself cannot substitute for robust institutions for fiscal accountability, using digital payments creates a transparent, auditable transactional record, which has the potential to improve health system management, and reduce payment delays and leakage of funds.

The use of digital payments also has several potential benefits for workers. First, digital financial payments may facilitate payment processing, reduce payment delays and increase the likelihood that payments are complete. Delayed and incomplete payments are damaging to workforce morale and well-being, and represent obstacles to improving health system performance and reform.^18^ One analysis found that an average of one out of every 3 days over a 10-year study period was affected by disruptions in care attributable to health workers’ strikes in LMICs.^19^ Wage concerns were at issue in more than half of all observed health worker strikes, and a quarter raised concerns regarding delayed salary payments.^19^ Delayed and incomplete payments have also been cited by health workers as an explanation for the solicitation of informal payments from patients.^20^ Digital payments may also create a clearer link between workers’ actions and their pay, paving the way for incentive programmes to support service delivery. Improvements in processes and payment outcomes for workers may improve worker morale, performance, hours worked and retention, which could, in turn, lead to improvements in the delivery of high quality health services.

There is a lack of availability of systematic data surrounding the specific characteristics of community and facility-based health worker payment methods (ie, cash, bank transfers and mobile deposits) and the procedures needed to track work and receive approval for payment across different settings.^21^ Many cash transfer programmes during the COVID-19 pandemic allowed beneficiaries to choose between cash-based, bank-based and mobile-based payment modalities.^22^ However, this kind of flexibility in payment is relatively rare in the health sector, despite the growing popularity of mobile money systems particularly in sub-Saharan Africa.^23^ Moreover, cross-country data collection is needed to describe the implementation of payment schemes and to document the scope of payment delays and incomplete payments. This would allow for analysis of the relationship between payment challenges and worker performance, absenteeism and retention, which has primarily been explored in qualitative research.^5 19^ While easier work documentation processes and timely payment may improve morale, they may be insufficient to improve performance without social recognition, supportive supervision, peer support and other enablers. Furthermore, improvements in payment processes at lower levels may be insufficient to improve payment outcomes if significant bottlenecks occur in the distribution and allocation of funds from national and state-level authorities.

Our conceptual framework lays out the pathways through which payment digitisation could have positive impacts on the health system. Though our model presents linear relationships, there are likely to be more complex interplays in these relationships in the context of health systems as complex adaptive systems.^24^ Furthermore, there are critical gaps in evidence to support this model. Implementation research is needed to document barriers and facilitators of payment digitisation where efforts to digitise payments are currently occurring. This could also include an understanding of the potential implications of wage digitisation for equity and inclusion within the health sector. This is particularly important as evidence from digitial financial services research suggests important barriers to access among some groups (especially women)^25^ and in some less digitally connected areas. Evidence is needed on how to ensure that efforts to digitise health worker payments do not exclude health workers who have less access to digital financial tools.

We provide a conceptual model for evaluating the impact of digitising payments and outline critical evidence gaps in understanding payment processes. As programmes transition toward digital payments, in part spurred by the COVID-19 crisis, learning from these efforts will shed light on foundational questions about how to support health workers in delivering high quality healthcare.

## Data Availability

There are no data in this work.
